# Linking off-target kinase pharmacology to the differential cellular effects observed among PARP inhibitors

**DOI:** 10.18632/oncotarget.1814

**Published:** 2014-03-10

**Authors:** Albert A. Antolín, Jordi Mestres

**Affiliations:** ^1^ Systems Pharmacology, Research Program on Biomedical Informatics, IMIM Hospital del Mar Medical Research Institute and Universitat Pompeu Fabra, Doctor Aiguader 88, 08003 Barcelona, Catalonia, Spain

**Keywords:** PARP inhibitors, off-target pharmacology, kinase profiling, drug combinations, biomarkers

## Abstract

PARP inhibitors hold promise as a novel class of targeted anticancer drugs. However, their true mechanism of action is still not well understood following recent reports that show marked differences in cellular effects. Here, we demonstrate that three PARP drug candidates, namely, rucaparib, veliparib, and olaparib, have a clearly different *in vitro* affinity profile across a panel of diverse kinases selected using a computational approach that relates proteins by ligand similarity. In this respect, rucaparib inhibits nine kinases with micromolar affinity, including PIM1, PIM2, PRKD2, DYRK1A, CDK1, CDK9, HIPK2, CK2, and ALK. In contrast, olaparib does not inhibit any of the sixteen kinases tested. In between, veliparib inhibits only two, namely, PIM1 and CDK9. The differential kinase pharmacology observed among PARP inhibitors provides a plausible explanation to their different cellular effects and offers unexplored opportunities for this drug class, but alerts also on the risk associated to transferring directly both preclinical and clinical outcomes from one PARP drug candidate to another.

## INTRODUCTION

The evidence that a Poly(ADP-ribose)polymerase (PARP) inhibitor provided clinical benefit to patients carrying breast-cancer-associated BRCA1 or BRCA2 gene mutations was a breakthrough in cancer therapy [1] and boosted the initiation of clinical trials involving several other PARP inhibitors. However, despite the early enthusiasm, progress of PARP drug candidates to the clinic has been slower than expected [2]. After several setbacks, PARP inhibitors finally have advanced to Phase III clinical trials [3] in spite of the fact that their mechanism of action is still not fully understood [4]. In this respect, there is a growing body of evidence to suggest that PARP inhibitors may exert their therapeutic effect through slightly different mechanisms of action, which could explain why some patients without BRCA mutations respond also to treatment with some PARP inhibitors [3,4]. Several studies have shown recently that different PARP inhibitors, once perceived as equivalent within the same drug class, have significantly different cellular effects when used at micromolar concentrations [5-7]. In addition, unexpected differences between PARP inhibitors also emerge from a recent analysis of the genomic biomarkers of drug sensitivity in cancer cell lines [8]. As an example, EWS-FLI1 was found to be a sensitivity biomarker of rucaparib and olaparib but surprisingly not of veliparib [8]. All these differences cannot be explained on the basis of the relatively similar affinity profiles of PARP drug candidates across 13 members of the PARP family [9] and they are thus indicative of the potential involvement of off-target affinities for proteins beyond PARPs. Accordingly, gaining a deeper understanding on the pharmacology of PARP inhibitors beyond the PARP protein family is essential to understand the differences observed at the clinical, cellular, and biomarker levels.

**Figure 1:**
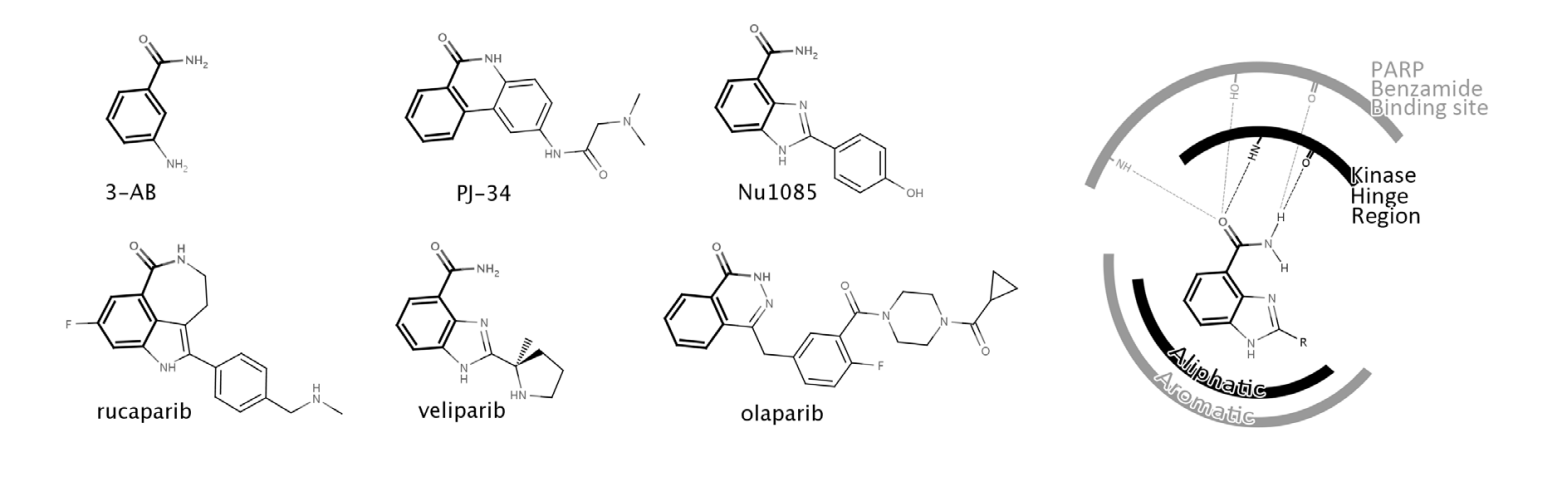
Chemical structures of PARP inhibitors including the PARP drug candidates rucaparib, veliparib and olaparib (left) The benzamide moiety that characterizes all PARP inhibitor structures is highlighted in bold. Schematic representation of the benzamide binding to both S6K1 kinase (PDB 4C35), depicted in black, and PARP-1 (PDB 2RD6), depicted in grey (right).

We previously reported that PJ34, an early chemical tool widely used to probe the biological function of PARP-1, is a micromolar inhibitor of PIM1 kinase (IC_50_ = 3.7 µM) [10]. The significance of this result lies in the fact that PIM1 is a confounding off-target in PARP biology, known to be involved in many processes relevant to cancer and thus likely to have synergistic effects with PARPs. Since the structure of PJ34, and of many of the PARP inhibitors currently in clinical trials, evolved from the most simple structure of 3-AB (Figure 1) [11], one may be tempted to speculate that PARP drug candidates may have also affinity for PIM1. Indeed, a close inspection to ChEMBL [12] revealed that PIM1 inhibition by rucaparib (CHEMBL1173055) had already been deposited in the database (K_i_ = 1.3 µM) from a large kinase profiling campaign [13]. However, this result has largely passed unnoticed [3], emphasizing the fact that depositing data in publicly available resources does not guarantee common awareness. Accordingly, we decided to investigate whether other PARP drug candidates have also *in vitro* affinity for PIM1 and explore further their potential off-target kinase pharmacology as a means to better understand their mechanism of action.

## RESULTS

**Figure 2:**
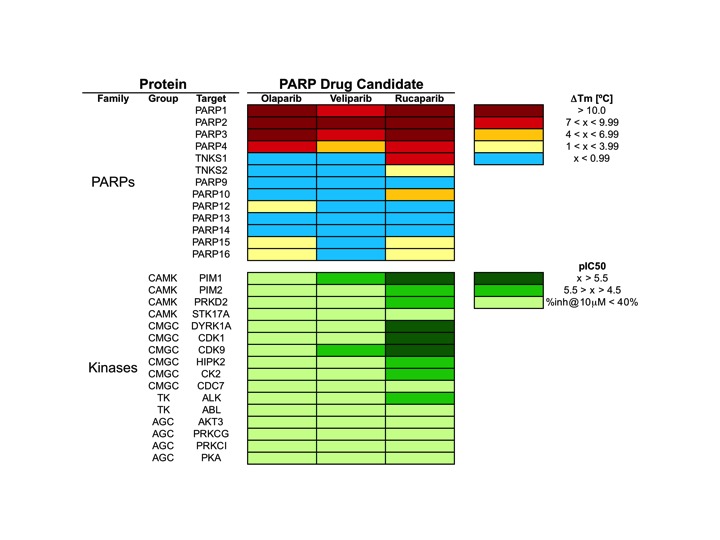
Pharmacological profile of olaparib, veliparib and rucaparib across 29 proteins, including 13 PARPs and 16 kinases PARP data is from Ref. (9); kinase data is from this work. Dose-response curves are available in the supplementary data for the 11 kinase interactions identified with pIC_50_ values above 4.5.

**Figure 3:**
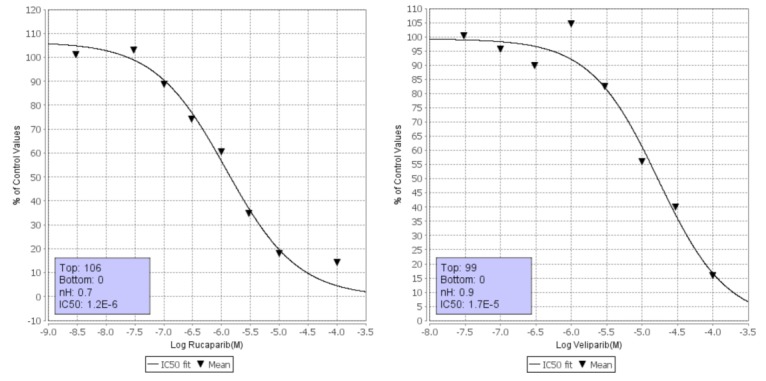
Dose-response curves of the *in vitro* affinity of rucaparib (left) and veliparib (right) with PIM1 kinase

The results of the *in vitro* kinase profiling clearly demonstrate that PARP drug candidates have different affinity for PIM1 and related kinases, as summarized in Figure 2 (dose-response curves available as supplementary data). For the sake of completeness, a recently published comprehensive comparison of the affinities of these drugs on 13 PARP family members is also included [9]. It is worth noting that both assays are not directly comparable in terms of affinity as the PARP profiling was done using differential scanning fluorimetry instead of inhibition. However, they enable us to comprehensively compare how these PARP drug candidates interact with kinases and PARPs. Above all, it is interesting to stress that while olaparib and rucaparib have a relatively similar affinity profile among the members of the PARP family, they differ significantly in their respective kinase profiles. As can be observed, while olaparib has no relevant affinities for any of the 16 protein kinases tested, rucaparib presents micromolar affinities (IC_50_ values) for 9 of them, namely, PIM1 (1.2 µM), PIM2 (7.7 µM), PRKD2 (9.7 µM), DYRK1A (1.4 µM), CDK1 (1.4 µM), CDK9 (2.7 µM), HIPK2 (4.4 µM), CK2 (7.8 µM), and ALK (18 µM). In this respect, olaparib appears to be a markedly more selective PARP inhibitor than rucaparib. In between, veliparib shows low micromolar affinities for PIM1 (17 µM) and CDK9 (8.2 µM). Dose-response curves of the *in vitro* binding affinity of rucaparib and veliparib for PIM1 kinase are shown in Figure 3. Remarkably, in line with the selection of 11 of those kinases by ligand similarity to PIM1, it is observed that the higher the affinity of the PARP inhibitor for PIM1, the higher the number of additional kinases to which the compound has affinity. Overall, the results presented here provide clear evidence that, at micromolar concentrations, confounding/synergistic effects from affinities of PARP inhibitors to various kinases deserve serious consideration.

Having confirmed that different PARP drug candidates are linked to essentially different kinase profiles, we wondered whether that could just be the tip of the iceberg. A recent HTS screening against S6K1 kinase surprisingly unraveled that a PARP inhibitor (Nu1085) (Figure 1) was also inhibiting S6K1 kinase with high affinity (IC_50_ = 0.56 µM) [14]. The crystallization of Nu1085 bound to S6K1 kinase [14] enabled us to compare how the benzamide common to all structures of PARP inhibitors (Figure 1) interacts with both kinases and PARPs, offering an explanation at a molecular level for the observed off-target kinase pharmacology of PARP inhibitors. As schematically illustrated in Figure 1, the benzamide group binds to the kinase hinge region, a highly conserved region among kinases located at the ATP binding site [14]. Moreover, the interactions of the benzamide are very similar in both PARP-1 and S6K1 hinge region (Figure 1). Therefore, PARP inhibitors might have a natural tendency to inhibit kinases due to the presence of this benzamide moiety in their structures. As the chemical structures grow from the hinge region to the gatekeeper residue (as is the case of olaparib), PARP inhibitors are likely to clash/interact with pockets on the back cleft of kinases and gain selectivity for PARPs over kinases [14]. This way, different PARP inhibitors will interact differently with kinases depending on their size and decoration, in line with the results reported here (Figure 2).

To strengthen this hypothesis, we searched ChEMBL [12] for dual PARP-kinase inhibitors and we found that 4 other PARP inhibitors are already reported to interact differently with many kinases [12,13]. One of these compounds, CHEMBL539474, inhibits both PARP-1 (Ki = 0.698 µM) and kinases known to synergize with PARP-1 with high affinity [15], namely, PLK1 (Ki = 0.079 µM) or GSK3A (Ki = 0.2 µM). We also used the webserver MANTRA to investigate the gene expression profile of the only PARP inhibitor present in the MANTRA database, 1,5-isoquinolinediol [16]. When the gene expression profile of 1,5-isoquinolinediol across 4 breast cancer cell lines is compared to the other 1300 drugs available in MANTRA, the most similar gene expression signature is the one corresponding to the EGFR tyrosine kinase drug gefitinib. This result highlights also that PARP and kinase inhibitors regulate genes in a similar way, suggesting that they may share mechanism-of-action targets. In light of all these findings, the kinase pharmacology of PARP inhibitors reported here is likely to expand as more PARP inhibitors are screened against larger panels of kinases.

## DISCUSSION

The differential kinase polypharmacology among PARP drug candidates offers a reasonable explanation for some of the differences observed in their cellular effects [5-7]. For example, the unique capacity of rucaparib to inhibit STAT3 phosphorilation at 5 µM [6] could be ascribed to its micromolar affinity for DYRK1A and/or CDK1, both direct phospohorilators of STAT3 [17,18]. In contrast, these kinases are not inhibited by olaparib or veliparib and, consequently, these drugs do not affect the phosphorilation state of STAT3. Similarly, CDK1 and PIM1 both regulate G2/M transition [18,19], providing a rational for the higher capacity of rucaparib to produce G2/M cell cycle arrest [6]. Moreover, kinase phosphorilation at the zinc-finger 1 domain of PARP-1 has been postulated as a regulatory mechanism to disrupt PARP-DNA binding [20], a plausible explanation to the different capacity of PARP inhibitors to trap PARP-1 at the DNA damage site at micromolar concentrations [7, 21]. Finally, it is worth stressing the low-affinity interaction identified between rucaparib and ALK (IC_50_ = 18 µM), which might partially explain the increased efficacy of rucaparib in cancer cells with alterations in ALK [8]. To the best of our knowledge, this represents the first example of a genomic biomarker of response being a confirmed off-target of the drug.

The experimental confirmation that PARP drug candidates have a unique and differential off-target profile across multiple kinases known to be involved in cancer-relevant processes provides a completely new perspective of PARP inhibitors in clinical trials. Some of the new affinities identified may offer opportunities for expanding the current clinical scope of PARP inhibitors, just like other off-targets have led to new indications of cancer drugs [22]. For instance, PIM1 overexpression in a number of hematopoietic cancers could promote the clinical investigation of rucaparib in acute myeloid leukemia [19]. In this sense, the recent profile of a drug panel across AML patient samples shows unexpected different sensitivity of different PARP drugs across samples, with some samples being sensitive only to rucaparib and others to olaparib [23]. Also, some of these kinases could help to identify the patient population that is responding to PARP inhibitors despite being BRCA mutation-negative [3], for example by including ALK as a biomarker of rucaparib response.

But most importantly, due to known synergistic effects between PARPs and kinases [15], compounds generally referred to as PARP inhibitors may not be considered as clinically equivalent anymore. This has significant implications for the direct transfer of conclusions derived from clinical data obtained using one PARP inhibitor to another one, with special impact in the combination of PARP and kinase inhibitors [24]. An example of this situation is the current clinical investigation of the potential synergistic anticancer effects between PARP-1 and CDK1 using veliparib and dinaciclib (clinical trial NCT01434316), whereas synergism between those two targets was validated in cellular studies using rucaparib and RO-3306 [25]. The fact that veliparib and rucaparib have a markedly different kinase profile (Figure 2), with only rucaparib directly inhibiting CDK1, warns on the extrapolation of the results from this clinical trial to PARP inhibitors other than veliparib itself. Beyond the combination with kinase inhibitors, PARP inhibitors have been combined with other drugs, chemotherapeutic agents, and radiation in a number of pre-clinical and clinical studies [26-28]. It is well known that the inhibition of different kinases, including PIM1 and CDKs, can induce sensitivity [29,30] or resistance [31] to some of those chemotherapeutic agents. Therefore, the off-target kinase pharmacology of PARP inhibitors could have also an effect on the sensitivity or resistance to chemotherapeutic agents used in combination. This possibility should now be taken seriously into consideration, in particular for combinations using rucaparib.

In spite of the biological relevance of the low-micromolar off-target affinities identified, one may argue that their true clinical significance is unclear due to the fact that the peak plasma concentration of PARP drugs is likely to be well below the IC_50_ values obtained for those kinases. For example, the highest peak concentration for a 50 mg single daily dose of veliparib is estimated to be around 1 µM [32]. Under these conditions, none of the two off-target affinities identified for veliparib (17 µM for PIM1 and 8.2 µM for CDK9) could be considered clinically relevant. However, a recent clinical study reported that a rather wide range of dose regimes is currently being explored in a phase I trial of veliparib, with maximum doses up to 400 mg twice daily [33]. With veliparib doses up to 8 times higher than the original dose, any off-target affinity close to 8 µM, such as the one for CDK9, may now become clinically relevant. In the case of rucaparib, its initial highest peak plasma concentration corresponding to a 40 mg single daily dose is estimated to be around 2 µM [34]. Under this regime, at least three kinases should be already considered clinically relevant off-targets for rucaparib, namely, PIM1, CDK1, and DYRK1A. However, in data presented at the last ASCO meeting [35], an ongoing phase I dose-escalation study of continuous oral rucaparib in patients with advanced solid tumors is using doses of up to 480 mg twice daily. Again, with rucaparib doses of up to 12 times higher than the original dose, all nine off-target affinities for kinases should be considered clinically relevant. It seems thus clear that off-target kinase affinities of PARP inhibitors should definitely be regarded as clinically relevant and thus be considered for establishing recommended phase II doses for rucaparib and veliparib.

We may have just scratched the surface of the off-target pharmacology linked to PARP inhibitors but we have learned enough to realize that they have a clear and differential kinase pharmacology beyond their primary PARP targets. Recent clinical trials are resurrecting the interest on PARP inhibitors, despite ignoring the existence of a wealth of additional interactions outside the PARP target space [3]. In the view of the data presented here, we urgently need a broader understanding of the mechanism of action of PARP inhibitors to guide their clinical development. Our results are indicative of the clear need for a wide pre-clinical target profiling of PARP inhibitors across at least a diversity panel of kinases to clarify whether the results from clinical studies on one PARP inhibitor can be transferred to other PARP inhibitors. What appeared as a single robust class of PARP inhibitors with similar pharmacological properties [3] should now be regarded as a promising set of compounds with high affinity for PARPs but linked also to a rich polypharmacology across multiple off-targets that makes them essentially unique and thus expands largely their potential therapeutic opportunities.

## METHODS

### Kinase Selection

Taking PIM1 as a reference kinase based on previous findings [10], we used a recently reported computational approach to organize proteins by ligand similarity [36] to identify 15 kinases for which more than 60% of their active ligands (pIC_50_ > 5) known in publicly available repositories [12] were also known to be active on PIM1. From these kinases, we selected the 11 that were available for screening at Cerep (www.cerep.fr). The selection included members of three different kinase groups distantly related by sequence (all having less than 20% sequence identity with PIM1), namely, CAMK (PIM2, STK17A), CMGC (DYRK1A, CDK9, HIPK2, CK2, and CDC7) and AGC (AKT3, PRKCG, PRKCI, and PKA). To this list, we added also two kinases belonging to the TK group that have been identified as biomarkers of PARP drug sensitivity [8] and shared ligands with PIM1, namely, ABL and ALK. The final list was complemented with two additional kinases reported to interact with rucaparib, namely, CDK1 and PRKD2 [12,13]. In the end, a total of 16 protein kinases were selected for a focused *in vitro* screening of PARP inhibitors.

### Kinase *in vitro* screening

Rucaparib, veliparib and olaparib were purchased from Selleckchem. Kinase in vitro assays were perfomed at Cerep (www.cerep.fr) by measuring the phosphorylation of appropriate peptide substrates by human recombinant enzymes and using FRET as a detection method (a detailed description of each kinase assay is available in online supplementary data).

## SUPPLEMENTARY INFORMATION AND FIGURES


