# Supplementary material for: Linking off-target kinase pharmacology to the differential cellular effects observed among PARP inhibitors
Source: Oncotarget. 2014 Mar 10;5(10):3023–8. doi: 10.18632/oncotarget.1814 (PMC4102788; doi:10.18632/oncotarget.1814)

## Supporting Data for:

# Linking off-target kinase pharmacology to the differential cellular effects observed among PARP inhibitors

Albert A. Antolín,[1] and Jordi Mestres[1*]

[1] Systems Pharmacology, Research Program on Biomedical Informatics, IMIM Hospital del Mar Medical Research Institute and Universitat Pompeu Fabra, Doctor Aiguader 88, 08003 Barcelona, Catalonia, Spain. * e-mail: jmestres@imim.es

**This file includes:**

Supplementary Methods

- Human kinase *in vitro* inhibition assays

Supplementary Results

- Kinase *in vitro* screening at 10μM
- Dose-response curves and $IC_{50}$s from the *in vitro* assays

# Supplementary Methods

## Human Pim1 and Pim2 kinase *in vitro* inhibition assays

All details for the Pim1 and Pim2 *in vitro* assays can be consulted in the Cerep Catalogue available online under references 2919 (745-P1) and 2920 (745-P2): http://www.cerep.fr/cerep/users/pages/catalog/Affiche_CondExp_Test.asp?test=2919, http://www.cerep.fr/cerep/users/pages/catalog/Affiche_CondExp_Test.asp?test=2920.

Evaluation of the effects of compounds on the activity of the human Pim1 and Pim2 kinases were quantified by measuring the phosphorylation of the substrate Ulight-CREBtide (CKRREILSRRPSYRK) using either Pim1 or Pim2 human recombinant enzymes expressed in insect cells and the LANCE® detection method[18,19].

The test compound, reference compound or water (control) are mixed with either Pim1 (4.08 ng) or Pim2 (6.36 ng) enzymes in a buffer containing 40 mM Hepes/Tris (pH 7.4), 0.8 mM EGTA/Tris, 8 mM MgCl2, 1.6 mM DTT and 0.008% Tween 20. Thereafter, the reaction is initiated by adding 25 nM of the substrate Ulight-CREBtide (CKRREILSRRPSYRK) and ATP (30 µM for Pim1 and 3 µM for Pim2), and the mixture is incubated for 60 min at room temperature. For control basal measurements, the enzyme is omitted from the reaction mixture. Following incubation, the reaction is stopped by adding 13 mM EDTA. After 5 min, the anti-phopho-CREB antibody labeled with europium chelate is added. After 60 more min, the fluorescence transfer is measured at λex=337 nm, λem=620 nm and λem=665 nm using a microplate reader (Envision, Perkin Elmer).

The enzyme activity is determined by dividing the signal measured at λem=665 nm by that measured at λem=620 nm (ratio). The results are expressed as a percent inhibition of the control enzyme activity. The standard inhibitory reference compound is staurosporine, which is tested in each experiment at several concentrations to obtain an inhibition curve from which its $IC_{50}$ value is calculated.

The $IC_{50}$ values (concentration causing a half-maximal inhibition of control specific activity) and Hill coefficients (nH) were determined by non-linear regression analysis of the inhibition curves generated with mean replicate values using Hill equation curve fitting (Y = D + [(A – D)/(1 + (C/C50)nH)], where Y = specific activity, D = minimum specific activity, A = maximum specific activity, C = compound concentration, C50 = $IC_{50}$, and nH = slope factor). This analysis was performed using a

software developed at Cerep (Hill software) and validated by comparison with data generated by the commercial software SigmaPlot® 4.0 for Windows® (© 1997 by SPSS Inc.).

## Human PRKD2 kinase *in vitro* inhibition assay

All details for the PRKD2 *in vitro* assay can be consulted in the Cerep Catalogue available online under references 1729 (742-pkd2): http://www.cerep.fr/cerep/users/pages/catalog/Affiche_CondExp_Test.asp?test=1729

Evaluation of the effects of compounds on the activity of the human PKD2 quantified by measuring the phosphorylation of the substrate biotinyl-βAβAβAKKKVSRSGLYRSPSMPENLNRPR using a human recombinant enzyme expressed in insect cells and the HTRF detection method.

The test compound, reference compound or water (control) are preincubated for 5 min at room temperature with the enzyme (3 ng) in a buffer containing 50 mM Hepes/NaOH (pH 7.4), 2 mM $MgCl_2$, 1 mM DTT, 40 µM $Na_3VO_4$ and 0.005% Tween 20. Thereafter, the reaction is initiated by adding 25 nM of the substrate biotinyl-βAβAβAKKKVSRSGLYRSPSMPENLNRPR and 30 µM ATP, and the mixture is incubated for 30 min at room temperature. For control basal measurements, the enzyme is omitted from the reaction mixture. Following incubation, the reaction is stopped by adding 33 mM EDTA. The fluorescence acceptor (XL665-labeled streptavidine) and the fluorescence donor (anti-phospho-Ser-K antibody labeled with europium cryptate) are then added. After 60 min, the fluorescence transfer is measured at λex=337 nm, λem=620 and λem=665 nm using a microplate reader (Rubystar, BMG). The enzyme activity is determined by dividing the signal measured at λem=665 nm by that measured at λem=620 nm (ratio). The results are expressed as a percent inhibition of the control enzyme activity. The standard inhibitory reference compound is staurosporine, which is tested in each experiment at several concentrations to obtain an inhibition curve from which its $IC_{50}$ value is calculated.

The $IC_{50}$ values (concentration causing a half-maximal inhibition of control specific activity) and Hill coefficients (nH) were determined by non-linear regression analysis of the inhibition curves generated with mean replicate values using Hill equation curve fitting (Y = D + [(A – D)/(1 + (C/C50)nH)], where Y = specific activity, D = minimum specific activity, A = maximum specific activity, C = compound concentration, C50 = $IC_{50}$, and nH = slope factor). This analysis was performed using a

software developed at Cerep (Hill software) and validated by comparison with data generated by the commercial software SigmaPlot® 4.0 for Windows® (© 1997 by SPSS Inc.).

### Human STK17A kinase *in vitro* inhibition assay

All details for the DRAK1 (STK17A) *in vitro* assay can be consulted in the Cerep Catalogue available online under references 2930 (745-D1):
http://www.cerep.fr/cerep/users/pages/catalog/Affiche_CondExp_Test.asp?test=2930

Evaluation of the effects of compounds on the activity of the human DRAK1 quantified by measuring the phosphorylation of the substrate Ulight-ARTKQTARKSTGGKAPRKQLAGCG (histone H3) using a human recombinant enzyme and the LANCE® detection method.

The test compound, reference compound or water (control) are mixed with the enzyme (5.552 ng) in a buffer containing 40 mM Hepes/Tris (pH 7.4), 0.8 mM EGTA/Tris, 8 mM $MgCl_2$, 1.6 mM DTT and 0.008% Tween 20. Thereafter, the reaction is initiated by adding 50 nM of the substrate Ulight ARTKQTARKSTGGKAPRKQLAGCG (histone H3) and 10 µM ATP, and the mixture is incubated for 120 min at room temperature. For control basal measurements, the enzyme is omitted from the reaction mixture. Following incubation, the reaction is stopped by adding 13 mM EDTA. After 5 min, the anti-phopho-histone H3 antibody labeled with europium chelate is added. After 60 more min, the fluorescence transfer is measured at λex=337 nm, λem=620 nm and λem=665 nm using a microplate reader (Envision, Perkin Elmer). The enzyme activity is determined by dividing the signal measured at λem=665 nm by that measured at λem=620 nm (ratio). The results are expressed as a percent inhibition of the control enzyme activity. The standard inhibitory reference compound is staurosporine, which is tested in each experiment at several concentrations to obtain an inhibition curve from which its $IC_{50}$ value is calculated.

### Human CDK1 kinase *in vitro* inhibition assay

All details for the CDC2/CDK1 *in vitro* assay can be consulted in the Cerep Catalogue available online under references 2875 (781-cdc2):
http://www.cerep.fr/cerep/users/pages/catalog/Affiche_CondExp_Test.asp?test=2875

Evaluation of the effects of compounds on the activity of the human CDC2/CDK1 quantified by measuring the phosphorylation of the substrate Ulight-CFFKNIVTPRTPPPSQGK-amide (MBP) using a human recombinant enzyme expressed in insect cells and the LANCE® detection method.

The test compound, reference compound or water (control) are mixed with the enzyme (2.28 ng) in a buffer containing 40 mM Hepes/Tris(pH 7.4), 0.8 mM EGTA/Tris, 8 mM $MgCl_2$, 1.6 mM DTT and 0.008% Tween 20. Thereafter, the reaction is initiated by adding 100 nM of the substrate Ulight-CFFKNIVTPRTPPPSQGK-amide (MBP) and 10 µM ATP, and the mixture is incubated for 15 min at room temperature. For control basal measurements, the enzyme is omitted from the reaction mixture. Following incubation, the reaction is stopped by adding 13 mM EDTA. After 5 min, the anti-phospho-MBP antibody labeled with europium chelate is added. After 60 more min, the fluorescence transfer is measured at λex=337 nm, λem=620 nm and λem=665 nm using a microplate reader (Envision, Perkin Elmer). The enzyme activity is determined by dividing the signal measured at λem=665 nm by that measured at λem=620 nm (ratio). The results are expressed as a percent inhibition of the control enzyme activity. The standard inhibitory reference compound is staurosporine, which is tested in each experiment at several concentrations to obtain an inhibition curve from which its $IC_{50}$ value is calculated.

The $IC_{50}$ values (concentration causing a half-maximal inhibition of control specific activity) and Hill coefficients (nH) were determined by non-linear regression analysis of the inhibition curves generated with mean replicate values using Hill equation curve fitting (Y = D + [(A – D)/(1 + (C/C50)nH)], where Y = specific activity, D = minimum specific activity, A = maximum specific activity, C = compound concentration, C50 = $IC_{50}$, and nH = slope factor). This analysis was performed using a software developed at Cerep (Hill software) and validated by comparison with data generated by the commercial software SigmaPlot® 4.0 for Windows® (© 1997 by SPSS Inc.).

## Human DYRK1A kinase *in vitro* inhibition assay

All details for the DYRK1A *in vitro* assay can be consulted in the Cerep Catalogue available online under references 2781 (781-dyrk1a): http://www.cerep.fr/cerep/users/pages/catalog/Affiche_CondExp_Test.asp?test=2781

Evaluation of the effects of compounds on the activity of the human DYRK1a quantified by measuring the phosphorylation of the substrate Ulight-CFFKNIVTPRTPPPSQGK-amide (MBP) using a human recombinant enzyme and the LANCE® detection method.

The test compound, reference compound or water (control) are mixed with the enzyme (11.2 ng) in a buffer containing 40 mM Hepes/Tris(pH 7.4), 0.8 mM EGTA/Tris, 8 mM $MgCl_2$, 1.6 mM DTT and 0.008% Tween 20. Thereafter, the reaction is initiated by adding 100 nM of the substrate Ulight-CFFKNIVTPRTPPPSQGK-amide (MBP) and 10 μM ATP, and the mixture is incubated for 30 min at room temperature. For control basal measurements, the enzyme is omitted from the reaction mixture. Following incubation, the reaction is stopped by adding 13 mM EDTA. After 5 min, the anti-phospho-MBP antibody labeled with europium chelate is added. After 60 more min, the fluorescence transfer is measured at λex=337 nm, λem=620 nm and λem=665 nm using a microplate reader (Envision, Perkin Elmer). The enzyme activity is determined by dividing the signal measured at λem=665 nm by that measured at λem=620 nm (ratio). The results are expressed as a percent inhibition of the control enzyme activity. The standard inhibitory reference compound is staurosporine, which is tested in each experiment at several concentrations to obtain an inhibition curve from which its $IC_{50}$ value is calculated.

The $IC_{50}$ values (concentration causing a half-maximal inhibition of control specific activity) and Hill coefficients (nH) were determined by non-linear regression analysis of the inhibition curves generated with mean replicate values using Hill equation curve fitting (Y = D + [(A – D)/(1 + (C/C50)nH)], where Y = specific activity, D = minimum specific activity, A = maximum specific activity, C = compound concentration, C50 = $IC_{50}$, and nH = slope factor). This analysis was performed using a software developed at Cerep (Hill software) and validated by comparison with data generated by the commercial software SigmaPlot® 4.0 for Windows® (© 1997 by SPSS Inc.).

## Human CDK9 kinase *in vitro* inhibition assay

All details for the CDK9 *in vitro* assay can be consulted in the Cerep Catalogue available online under references 2912 (781-cdk9): http://www.cerep.fr/cerep/users/pages/catalog/Affiche_CondExp_Test.asp?test=2912

Evaluation of the effects of compounds on the activity of the human CDK9 quantified by measuring the phosphorylation of the substrate Ulight-CFFKNIVTPRTPPPSQGK-amide (MBP) using a human recombinant enzyme expressed in Sf9 cells and the LANCE® detection method.

The test compound, reference compound or water (control) are mixed with the enzyme (21.72 ng) in a buffer containing 40 mM Hepes/Tris(pH 7.4), 0.8 mM EGTA/Tris, 8 mM $MgCl_2$, 1.6 mM DTT and 0.008% Tween 20. Thereafter, the reaction is initiated by adding 100 nM of the substrate Ulight-CFFKNIVTPRTPPPSQGK-amide (MBP) and 10 µM ATP, and the mixture is incubated for 90 min at room temperature. For control basal measurements, the enzyme is omitted from the reaction mixture. Following incubation, the reaction is stopped by adding 13 mM EDTA. After 5 min, the anti-phospho-MBP antibody labeled with europium chelate is added. After 60 more min, the fluorescence transfer is measured at λex=337 nm, λem=620 nm and λem=665 nm using a microplate reader (Envision, Perkin Elmer). The enzyme activity is determined by dividing the signal measured at λem=665 nm by that measured at λem=620 nm (ratio). The results are expressed as a percent inhibition of the control enzyme activity. The standard inhibitory reference compound is staurosporine, which is tested in each experiment at several concentrations to obtain an inhibition curve from which its $IC_{50}$ value is calculated.

The $IC_{50}$ values (concentration causing a half-maximal inhibition of control specific activity) and Hill coefficients (nH) were determined by non-linear regression analysis of the inhibition curves generated with mean replicate values using Hill equation curve fitting (Y = D + [(A – D)/(1 + (C/C50)nH)], where Y = specific activity, D = minimum specific activity, A = maximum specific activity, C = compound concentration, C50 = $IC_{50}$, and nH = slope factor). This analysis was performed using a software developed at Cerep (Hill software) and validated by comparison with data generated by the commercial software SigmaPlot® 4.0 for Windows® (© 1997 by SPSS Inc.).

## Human HIPK2 kinase *in vitro* inhibition assay

All details for the HIPK2 *in vitro* assay can be consulted in the Cerep Catalogue available online under references 2915 (781-H2): http://www.cerep.fr/cerep/users/pages/catalog/Affiche_CondExp_Test.asp?test=2915

Evaluation of the effects of compounds on the activity of the human HIPK2 quantified by measuring the phosphorylation of the substrate Ulight-CFFKNIVTPRTPPPSQGK-amide (MBP) using a human recombinant enzyme and the LANCE® detection method.

The test compound, reference compound or water (control) are mixed with the enzyme (3.16 ng) in a buffer containing 40 mM Hepes/Tris(pH 7.4), 0.8 mM EGTA/Tris, 8 mM $MgCl_2$, 1.6 mM DTT and 0.008% Tween 20. Thereafter, the reaction is initiated by adding 50 nM of the substrate Ulight-CFFKNIVTPRTPPPSQGK-amide (MBP) and 10 µM ATP, and the mixture is incubated for 30 min at room temperature. For control basal measurements, the enzyme is omitted from the reaction mixture. Following incubation, the reaction is stopped by adding 13 mM EDTA. After 5 min, the anti-phospho-MBP antibody labeled with europium chelate is added. After 60 more min, the fluorescence transfer is measured at λex=337 nm, λem=620 nm and λem=665 nm using a microplate reader (Envision, Perkin Elmer). The enzyme activity is determined by dividing the signal measured at λem=665 nm by that measured at λem=620 nm (ratio). The results are expressed as a percent inhibition of the control enzyme activity. The standard inhibitory reference compound is staurosporine, which is tested in each experiment at several concentrations to obtain an inhibition curve from which its $IC_{50}$ value is calculated.

The $IC_{50}$ values (concentration causing a half-maximal inhibition of control specific activity) and Hill coefficients (nH) were determined by non-linear regression analysis of the inhibition curves generated with mean replicate values using Hill equation curve fitting (Y = D + [(A – D)/(1 + (C/C50)nH)], where Y = specific activity, D = minimum specific activity, A = maximum specific activity, C = compound concentration, C50 = $IC_{50}$, and nH = slope factor). This analysis was performed using a software developed at Cerep (Hill software) and validated by comparison with data generated by the commercial software SigmaPlot® 4.0 for Windows® (© 1997 by SPSS Inc.).

## Human CK2 kinase *in vitro* inhibition assay

All details for the CK2 (casein kinase 2) *in vitro* assay can be consulted in the Cerep Catalogue available online under references 2913 (781-CK2): http://www.cerep.fr/cerep/users/pages/catalog/Affiche_CondExp_Test.asp?test=2913

Evaluation of the effects of compounds on the activity of the human CK2 quantified by measuring the phosphorylation of the substrate Ulight-IkappaB-alpha using a human recombinant enzyme expressed in *E. coli* and the LANCE® detection method.

The test compound, reference compound or water (control) are mixed with the enzyme (14 ng) in a buffer containing 40 mM Hepes/Tris (pH 7.4), 0.8 mM EGTA/Tris, 8 mM $MgCl_2$, 3.6 mM DTT and 0.008% Tween 20. Thereafter, the reaction is initiated by adding 100 nM of the substrate Ulight-IkappaB-alpha and 500 nM ATP, and the mixture is incubated for 60 min at room temperature. For control basal measurements, the enzyme is omitted from the reaction mixture. Following incubation, the reaction is stopped by adding 13 mM EDTA. After 5 min, the anti-phopho-IkappaB-alpha antibody labeled with europium chelate is added. After 60 more min, the fluorescence transfer is measured at λex=337 nm, λem=620 nm and λem=665 nm using a microplate reader (Envision, Perkin Elmer). The enzyme activity is determined by dividing the signal measured at λem=665 nm by that measured at λem=620 nm (ratio). The results are expressed as a percent inhibition of the control enzyme activity. The standard inhibitory reference compound is heparin, which is tested in each experiment at several concentrations to obtain an inhibition curve from which its $IC_{50}$ value is calculated.

The $IC_{50}$ values (concentration causing a half-maximal inhibition of control specific activity) and Hill coefficients (nH) were determined by non-linear regression analysis of the inhibition curves generated with mean replicate values using Hill equation curve fitting (Y = D + [(A – D)/(1 + (C/C50)nH)], where Y = specific activity, D = minimum specific activity, A = maximum specific activity, C = compound concentration, C50 = $IC_{50}$, and nH = slope factor). This analysis was performed using a software developed at Cerep (Hill software) and validated by comparison with data generated by the commercial software SigmaPlot® 4.0 for Windows® (© 1997 by SPSS Inc.).

## Human CDC7 kinase *in vitro* inhibition assay

All details for the CDC7 *in vitro* assay can be consulted in the Cerep Catalogue available online under references 2764 (749-cdc7): http://www.cerep.fr/cerep/users/pages/catalog/Affiche_CondExp_Test.asp?test=2764

Evaluation of the effects of compounds on the activity of the human CDC7/ASK quantified by measuring the phosphorylation of the substrate Ulight-ARTKQTARKSTGGKAPRKQLAGCG (histone H3) using a human recombinant enzyme and the LANCE® detection method.

The test compound, reference compound or water (control) are mixed with the enzyme (9.2 ng) in a buffer containing 40 mM Hepes/Tris (pH 7.4), 0.8 mM EGTA/Tris, 8 mM $MgCl_2$, 1.6 mM DTT and 0.008% Tween 20. Thereafter, the reaction is initiated by adding 50 nM of the substrate Ulight-ARTKQTARKSTGGKAPRKQLAGCG (histone H3) and 10 µM ATP, and the mixture is incubated for 60 min at room temperature. For control basal measurements, the enzyme is omitted from the reaction mixture. Following incubation, the reaction is stopped by adding 13 mM EDTA. After 5 min, the anti-phopho-histone H3 antibody labeled with europium chelate is added. After 60 more min, the fluorescence transfer is measured at λex=337 nm, λem=620 nm and λem=665 nm using a microplate reader (Envision, Perkin Elmer). The enzyme activity is determined by dividing the signal measured at λem=665 nm by that measured at λem=620 nm (ratio). The results are expressed as a percent inhibition of the control enzyme activity. The standard inhibitory reference compound is staurosporine, which is tested in each experiment at several concentrations to obtain an inhibition curve from which its $IC_{50}$ value is calculated.

## Human ALK kinase *in vitro* inhibition assay

All details for the ALK *in vitro* assay can be consulted in the Cerep Catalogue available online under references 2678 (768-alk): http://www.cerep.fr/cerep/users/pages/catalog/Affiche_CondExp_Test.asp?test=2678

Evaluation of the effects of compounds on the activity of the human ALK quantified by measuring the phosphorylation of the substrate Ulight-CKKSRGDYMTMQIG (IRS-1) using a human recombinant enzyme and the LANCE® detection method.

The test compound, reference compound or water (control) are mixed with the enzyme (1.2 ng) in a buffer containing 40 mM Hepes/Tris (pH 7.4), 0.8 mM EGTA/Tris, 8 mM $MgCl_2$, 1.6 mM DTT and 0.008% Tween 20. Thereafter, the reaction is initiated by adding 150 nM of the substrate Ulight-CKKSRGDYMTMQIG (IRS-1) and 10 µM ATP, and the mixture is incubated for 60 min at room temperature. For control basal measurements, the enzyme is omitted from the reaction mixture. Following incubation, the reaction is stopped by adding 13 mM EDTA. After 5 min, the anti-phopho-PT66 antibody labeled with europium chelate is added. After 60 more min, the fluorescence transfer is measured at λex=337 nm, λem=620 nm and λem=665 nm using a microplate reader (Envision, Perkin Elmer). The enzyme activity is determined by dividing the signal measured at λem=665 nm by that measured at λem=620 nm (ratio). The results are expressed as a percent inhibition of the control enzyme activity. The standard inhibitory reference compound is staurosporine, which is tested in each experiment at several concentrations to obtain an inhibition curve from which its $IC_{50}$ value is calculated.

The $IC_{50}$ values (concentration causing a half-maximal inhibition of control specific activity) and Hill coefficients (nH) were determined by non-linear regression analysis of the inhibition curves generated with mean replicate values using Hill equation curve fitting (Y = D + [(A – D)/(1 + (C/C50)nH)], where Y = specific activity, D = minimum specific activity, A = maximum specific activity, C = compound concentration, C50 = $IC_{50}$, and nH = slope factor). This analysis was performed using a software developed at Cerep (Hill software) and validated by comparison with data generated by the commercial software SigmaPlot® 4.0 for Windows® (© 1997 by SPSS Inc.).

## Human ABL kinase *in vitro* inhibition assay

All details for the ABL *in vitro* assay can be consulted in the Cerep Catalogue available online under references 3056 (781-ablh):
http://www.cerep.fr/cerep/users/pages/catalog/Affiche_CondExp_Test.asp?test=3056

Evaluation of the effects of compounds on the activity of the human Abl kinase quantified by measuring the phosphorylation of the substrate Ulight-TK peptide using a human recombinant enzyme expressed in insect cells and the LANCE® detection method.

The test compound, reference compound or water (control) are mixed with the enzyme (about 0.4 ng) in a buffer containing 40 mM Hepes/Tris (pH 7.4), 0.8 mM EGTA/Tris, 8 mM $MgCl_2$, 1.6 mM DTT and 0.008% Tween 20. Thereafter, the reaction is initiated by adding 100 nM of the substrate Ulight-TK peptide and 10 µM ATP, and the mixture is incubated for 60 min at room temperature. For control basal measurements, the enzyme is omitted from the reaction mixture. Following incubation, the reaction is stopped by adding 13 mM EDTA. After 5 min, the anti-phospho-PT66 antibody labeled with europium chelate is added. After 60 more min, the fluorescence transfer is measured at λex=337 nm, λem=620 nm and λem=665 nm using a microplate reader (Envision, Perkin Elmer). The enzyme activity is determined by dividing the signal measured at λem=665 nm by that measured at λem=620 nm (ratio). The results are expressed as a percent inhibition of the control enzyme activity. The standard inhibitory reference compound is staurosporine, which is tested in each experiment at several concentrations to obtain an inhibition curve from which its $IC_{50}$ value is calculated.

## Human AKT3 kinase *in vitro* inhibition assay

All details for the AKT3 *in vitro* assay can be consulted in the Cerep Catalogue available online under references 2925 (706-akt3):
http://www.cerep.fr/cerep/users/pages/catalog/Affiche_CondExp_Test.asp?test=2925

Evaluation of the effects of compounds on the activity of the human Akt3/PKBγ quantified by measuring the phosphorylation of the substrate Ulight-RRRSLLE (PLK) using a human recombinant enzyme and the LANCE® detection method.

The test compound, reference compound or water (control) are mixed with the enzyme (2.8 ng) in a buffer containing 40 mM Hepes/Tris(pH 7.4), 0.8 mM EGTA/Tris, 8 mM $MgCl_2$, 1.6 mM DTT and 0.008% Tween 20. Thereafter, the reaction is initiated by adding 50 nM of the substrate Ulight-RRRSLLE (PLK) and 10 µM ATP, and the mixture is incubated for 30 min at room temperature. For control basal measurements, the enzyme is omitted from the reaction mixture. Following incubation, the reaction is stopped by adding 13 mM EDTA. After 5 min, the anti-phospho-PLK antibody labeled with europium chelate is added. After 60 more min, the fluorescence transfer is measured at λex=337 nm, λem=620 nm and λem=665 nm using a microplate reader (Envision,

Perkin Elmer). The enzyme activity is determined by dividing the signal measured at λem=665 nm by that measured at λem=620 nm (ratio). The results are expressed as a percent inhibition of the control enzyme activity. The standard inhibitory reference compound is staurosporine, which is tested in each experiment at several concentrations to obtain an inhibition curve from which its $IC_{50}$ value is calculated.

### Human PRKCG kinase *in vitro* inhibition assay

All details for the PRKCG (PKCγ) *in vitro* assay can be consulted in the Cerep Catalogue available online under references 0350 (705-gh): http://www.cerep.fr/cerep/users/pages/catalog/Affiche_CondExp_Test.asp?test=350

Evaluation of the effects of compounds on the activity of the human PKCγ quantified by measuring the phosphorylation of the substrate biotinyl-βAβAβAKIQASFRGHMARKK using a human recombinant enzyme expressed in insect cells and the HTRF detection method.

The test compound, reference compound or water (control) are preincubated for 5 min at room temperature with the enzyme (1 ng) in a buffer containing 50 mM Hepes/NaOH (pH 7.4), 1 mM $MgCl_2$, 0.72 mM $CaCl_2$, 1 mM DTT, 40 µM $Na_3VO_4$, 10 µM PMA, 0.005% Tween 20, and 0.014% phosphatidyl-serine. Thereafter, the reaction is initiated by adding 500 nM of the substrate biotinyl-βAβAβAKIQASFRGHMARKK and 1 µM ATP, and the mixture is incubated for 30 min at room temperature. For control basal measurements, the enzyme is omitted from the reaction mixture. Following incubation, the reaction is stopped by adding 33 mM EDTA. The fluorescence acceptor (XL665-labeled streptavidine) and the fluorescence donor (anti-phospho-Creb-K antibody labeled with europium cryptate) are then added. After 60 min, the fluorescence transfer is measured at λex=337 nm, λem=620 and λem=665 nm using a microplate reader (Rubystar, BMG). The enzyme activity is determined by dividing the signal measured at λem=665 nm by that measured at λem=620 nm (ratio). The results are expressed as a percent inhibition of the control enzyme activity. The standard inhibitory reference compound is Bis 10, which is tested in each experiment at several concentrations to obtain an inhibition curve from which its $IC_{50}$ value is calculated.

## Human PRKCI kinase *in vitro* inhibition assay

All details for the PRKCI (PKCι) *in vitro* assay can be consulted in the Cerep Catalogue available online under references 2121 (705-ih):
http://www.cerep.fr/cerep/users/pages/catalog/Affiche_CondExp_Test.asp?test=2121

Evaluation of the effects of compounds on the activity of the human PKCι quantified by measuring the phosphorylation of the substrate biotinyl-βAβAβAKIQASFRGHMARKK using a human recombinant enzyme expressed in insect cells and the HTRF detection method.

The test compound, reference compound or water (control) are preincubated for 5 min at room temperature with the enzyme (5 ng) in a buffer containing 50 mM Hepes/NaOH (pH 7.4), 5 mM $MgCl_2$, 1 mM DTT, 40 µM $Na_3VO_4$ and 0.005% Tween 20. Thereafter, the reaction is initiated by adding 200 nM of the substrate biotinyl-βAβAβAKIQASFRGHMARKK and 2 µM ATP, and the mixture is incubated for 30 min at room temperature. For control basal measurements, the enzyme is omitted from the reaction mixture. Following incubation, the reaction is stopped by adding 33 mM EDTA. The fluorescence acceptor (XL665-labeled streptavidine) and the fluorescence donor (anti-phospho-Creb K antibody labeled with europium cryptate) are then added. After 60 min, the fluorescence transfer is measured at λex=337 nm, λem=620 and λem=665 nm using a microplate reader (Rubystar, BMG). The enzyme activity is determined by dividing the signal measured at λem=665 nm by that measured at λem=620 nm (ratio).The results are expressed as a percent inhibition of the control enzyme activity. The standard inhibitory reference compound is Bis 10, which is tested in each experiment at several concentrations to obtain an inhibition curve from which its $IC_{50}$ value is calculated.

## Human PKA kinase *in vitro* inhibition assay

All details for the PKA *in vitro* assay can be consulted in the Cerep Catalogue available online under references 2927 (706-pka):
http://www.cerep.fr/cerep/users/pages/catalog/Affiche_CondExp_Test.asp?test=2927

Evaluation of the effects of compounds on the activity of the human PKA quantified by measuring the phosphorylation of the substrate Ulight-

RRRSLLE (PLK) using a human recombinant enzyme expressed in *E. coli* and the LANCE® detection method.

The test compound, reference compound or water (control) are mixed with the enzyme (0.044 ng) in a buffer containing 40 mM Hepes/Tris(pH 7.4), 0.8 mM EGTA/Tris, 8 mM $MgCl_2$, 1.6 mM DTT and 0.008% Tween 20. Thereafter, the reaction is initiated by adding 50 nM of the substrate Ulight-RRRSLLE (PLK) and 1 μM ATP, and the mixture is incubated for 10 min at room temperature. For control basal measurements, the enzyme is omitted from the reaction mixture. Following incubation, the reaction is stopped by adding 13 mM EDTA. After 5 min, the anti-phospho-PLK antibody labeled with europium chelate is added. After 60 more min, the fluorescence transfer is measured at λex=337 nm, λem=620 nm and λem=665 nm using a microplate reader (Envision, Perkin Elmer). The enzyme activity is determined by dividing the signal measured at λem=665 nm by that measured at λem=620 nm (ratio). The results are expressed as a percent inhibition of the control enzyme activity. The standard inhibitory reference compound is staurosporine, which is tested in each experiment at several concentrations to obtain an inhibition curve from which its $IC_{50}$ value is calculated.

# Supplementary Results

## Kinase *in vitro* screening at 10μM

**Supplementary Table 1** provides the raw data, in duplicate, for the interaction of olaparib, veliparib and rucaparib with the 16 kinases analyzed. Values are reported as percent inhibition of the control enzyme activity at 10μM drug concentration.

| Cerep Compound I.D. | Client Compound I.D. | Test Concentration | % Inhibition of control values 1st | 2nd | Mean |
|---|---|---|---|---|---|
| **Abl kinase *(h)*** | | | | | |
| 100007029-1 | Olaparib | 1.0E-05 M | -7.0 | -2.2 | -4.6 |
| 100007029-2 | Veliparib | 1.0E-05 M | -9.9 | 2.7 | -3.6 |
| 100007029-3 | Rucaparib | 1.0E-05 M | -41.7 | -6.6 | -24.2 |
| **ALK *(h)*** | | | | | |
| 100007029-1 | Olaparib | 1.0E-05 M | -4.0 | 4.6 | 0.3 |
| 100007029-2 | Veliparib | 1.0E-05 M | 6.7 | 4.1 | 5.4 |
| 100007029-3 | Rucaparib | 1.0E-05 M | 41.9 | 49.2 | 45.5 |
| **Akt3/PKBγ *(h)*** | | | | | |
| 100007029-1 | Olaparib | 1.0E-05 M | 4.7 | -2.4 | 1.1 |
| 100007029-2 | Veliparib | 1.0E-05 M | -6.3 | 1.7 | -2.3 |
| 100007029-3 | Rucaparib | 1.0E-05 M | 20.2 | 19.0 | 19.6 |
| **CDC2/CDK1 *(h)* (cycB)** | | | | | |
| 100007029-1 | Olaparib | 1.0E-05 M | 11.7 | -6.0 | 2.9 |
| 100007029-2 | Veliparib | 1.0E-05 M | 0.6 | 7.0 | 3.8 |
| 100007029-3 | Rucaparib | 1.0E-05 M | 74.1 | 68.6 | **71.3** |
| **CDC7 /ASK *(h)*** | | | | | |
| 100007029-1 | Olaparib | 1.0E-05 M | 0.6 | 0.7 | 0.6 |
| 100007029-2 | Veliparib | 1.0E-05 M | 11.7 | 11.3 | 11.5 |
| 100007029-3 | Rucaparib | 1.0E-05 M | 30.2 | 30.2 | 30.2 |
| **CDK9 *(h)* (cycT1)** | | | | | |
| 100007029-1 | Olaparib | 1.0E-05 M | 14.9 | 6.2 | 10.6 |
| 100007029-2 | Veliparib | 1.0E-05 M | 64.3 | 66.8 | **65.6** |
| 100007029-3 | Rucaparib | 1.0E-05 M | 75.2 | 80.1 | **77.7** |
| **CK2 *(h)* (casein kinase 2)** | | | | | |
| 100007029-1 | Olaparib | 1.0E-05 M | -0.7 | -2.2 | -1.4 |
| 100007029-2 | Veliparib | 1.0E-05 M | 19.2 | 15.9 | 17.6 |
| 100007029-3 | Rucaparib | 1.0E-05 M | 67.0 | 64.4 | **65.7** |
| **DRAK1 *(h)*** | | | | | |
| 100007029-1 | Olaparib | 1.0E-05 M | -5.3 | 1.6 | -1.8 |
| 100007029-2 | Veliparib | 1.0E-05 M | 6.6 | 5.4 | 6.0 |
| 100007029-3 | Rucaparib | 1.0E-05 M | 35.7 | 35.3 | 35.5 |
| **DYRK1a *(h)*** | | | | | |
| 100007029-1 | Olaparib | 1.0E-05 M | 7.8 | 19.2 | 13.5 |
| 100007029-2 | Veliparib | 1.0E-05 M | 11.9 | 6.6 | 9.3 |
| 100007029-3 | Rucaparib | 1.0E-05 M | 87.3 | 88.2 | **87.7** |
| **HIPK2 *(h)*** | | | | | |
| 100007029-1 | Olaparib | 1.0E-05 M | 5.7 | -8.8 | -1.5 |
| 100007029-2 | Veliparib | 1.0E-05 M | 6.8 | 2.9 | 4.8 |
| 100007029-3 | Rucaparib | 1.0E-05 M | 58.7 | 66.7 | **62.7** |
| **Pim1 kinase *(h)*** | | | | | |
| 100007029-1 | Olaparib | 1.0E-05 M | 3.0 | 12.0 | 7.5 |
| 100007029-2 | Veliparib | 1.0E-05 M | 45.2 | 46.9 | 46.0 |
| 100007029-3 | Rucaparib | 1.0E-05 M | 84.3 | 84.0 | **84.1** |
| **Pim2 kinase *(h)*** | | | | | |
| 100007029-1 | Olaparib | 1.0E-05 M | -34.0 | -23.7 | -28.8 |
| 100007029-2 | Veliparib | 1.0E-05 M | 14.2 | 20.2 | 17.2 |
| 100007029-3 | Rucaparib | 1.0E-05 M | 69.7 | 68.8 | **69.2** |
| **PKA *(h)*** | | | | | |
| 100007029-1 | Olaparib | 1.0E-05 M | 1.9 | 5.1 | 3.5 |
| 100007029-2 | Veliparib | 1.0E-05 M | 8.7 | 12.3 | 10.5 |
| 100007029-3 | Rucaparib | 1.0E-05 M | -1.3 | -1.4 | -1.4 |
| **PKCγ *(h)*** | | | | | |
| 100007029-1 | Olaparib | 1.0E-05 M | 3.1 | -0.7 | 1.2 |
| 100007029-2 | Veliparib | 1.0E-05 M | 5.2 | 6.1 | 5.6 |
| 100007029-3 | Rucaparib | 1.0E-05 M | 21.6 | 19.8 | 20.7 |
| **PKCι *(h)*** | | | | | |
| 100007029-1 | Olaparib | 1.0E-05 M | 6.0 | -24.9 | -9.5 |
| 100007029-2 | Veliparib | 1.0E-05 M | -9.3 | 2.5 | -3.4 |
| 100007029-3 | Rucaparib | 1.0E-05 M | 16.1 | 12.3 | 14.2 |
| **PKD2 *(h)*** | | | | | |
| 100007029-1 | Olaparib | 1.0E-05 M | -3.9 | 5.4 | 0.7 |
| 100007029-2 | Veliparib | 1.0E-05 M | 12.9 | 16.1 | 14.5 |
| 100007029-3 | Rucaparib | 1.0E-05 M | 54.9 | 57.3 | **56.1** |

## Dose-response curves and IC50s from the *in vitro* assays

**Supplementary Figure 1** provides the raw data, in duplicate, and the dose-response curve for the interaction of veliparib with human PIM1 kinase, as provided by the CRO (Cerep, http://www.cerep.fr/). Values are reported as percent inhibition of the control enzyme activity (see **Supplementary Methods** above). The $IC_{50}$ value calculated by non-linear regression analysis of the dose-response curve generated with mean replicate values using Hill equation curve fitting is also included as provided by Cerep.

| Cerep Compound I.D. | Client Compound I.D. | $IC_{50}$ (M) | nH | Test Concentration | % of Control Values 1st | 2nd | Mean |
|---|---|---|---|---|---|---|---|
| **Pim1 kinase *(h)*** | | | | | | | |
| 100007029-2 | Veliparib | 1.7E-05 M | 0.9 | 3.0E-08 M | 100.9 | 99.9 | 100.4 |
| | | | | 1.0E-07 M | 98.6 | 92.8 | 95.7 |
| | | | | 3.0E-07 M | 91.8 | 87.9 | 89.9 |
| | | | | 1.0E-06 M | 107.2 | 101.7 | 104.5 |
| | | | | 3.0E-06 M | 83.8 | 81.4 | 82.6 |
| | | | | 1.0E-05 M | 56.4 | 55.7 | 56.1 |
| | | | | 3.0E-05 M | 37.0 | 43.3 | 40.2 |
| | | | | 1.0E-04 M | 16.7 | 15.0 | 15.9 |

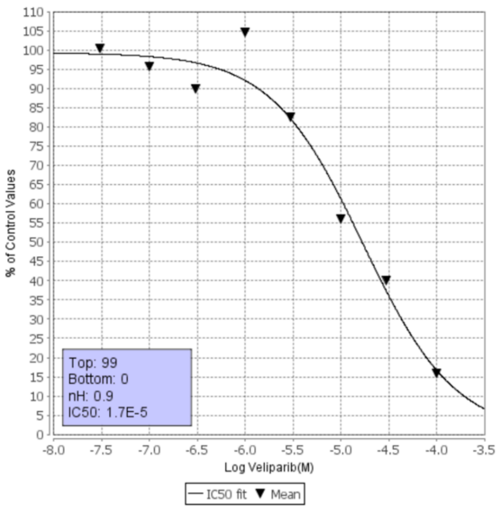

**Supplementary Figure 2** provides the raw data, in duplicate, and the dose-response curve for the interaction of veliparib with human CDK9 kinase, as provided by the CRO (Cerep, http://www.cerep.fr/). Values are reported as percent inhibition of the control enzyme activity (see **Supplementary Methods** above). The $IC_{50}$ value calculated by non-linear regression analysis of the dose-response curve generated with mean replicate values using Hill equation curve fitting is also included as provided by Cerep.

| Cerep Compound I.D. | Client Compound I.D. | $IC_{50}$ (M) | nH | Test Concentration | % of Control Values 1st | 2nd | Mean |
|---|---|---|---|---|---|---|---|
| **CDK9 *(h)* (cycT1)** | | | | | | | |
| 100007029-2 | Veliparib | 8.2E-06 M | 0.9 | 3.0E-08 M | 101.5 | 101.9 | 101.7 |
| | | | | 1.0E-07 M | 104.3 | 98.9 | 101.6 |
| | | | | 3.0E-07 M | 90.5 | 92.3 | 91.4 |
| | | | | 1.0E-06 M | 91.2 | 79.7 | 85.5 |
| | | | | 3.0E-06 M | 79.8 | 66.0 | 72.9 |
| | | | | 1.0E-05 M | 45.1 | 48.8 | 47.0 |
| | | | | 3.0E-05 M | 21.5 | 27.0 | 24.3 |
| | | | | 1.0E-04 M | 9.4 | 8.0 | 8.7 |

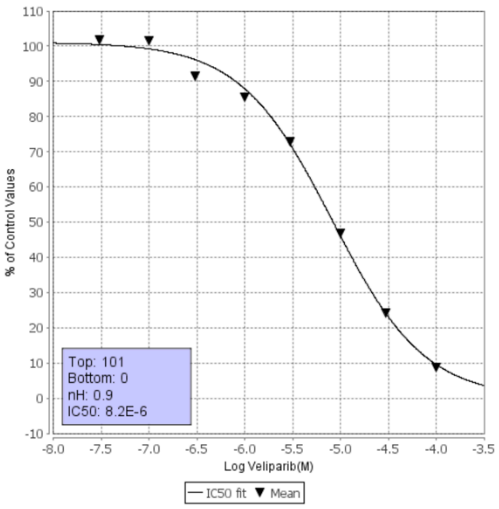

**Supplementary Figure 3** provides the raw data, in duplicate, and the dose-response curve for the interaction of rucaparib with human PIM1 kinase, as provided by the CRO (Cerep, http://www.cerep.fr/). Values are reported as percent inhibition of the control enzyme activity (see **Supplementary Methods** above). The $IC_{50}$ value calculated by non-linear regression analysis of the dose-response curve generated with mean replicate values using Hill equation curve fitting is also included as provided by Cerep.

| Cerep Compound I.D. | Client Compound I.D. | $IC_{50}$ (M) | nH | Test Concentration | % of Control Values 1st | 2nd | Mean |
|---|---|---|---|---|---|---|---|
| **Pim1 kinase *(h)*** | | | | | | | |
| 100007029-3 | Rucaparib | 1.2E-06 M | 0.7 | 3.0E-09 M | 102.3 | 99.9 | 101.1 |
| | | | | 3.0E-08 M | 111.7 | 94.5 | 103.1 |
| | | | | 1.0E-07 M | 91.1 | 86.2 | 88.7 |
| | | | | 3.0E-07 M | 74.0 | 74.7 | 74.3 |
| | | | | 1.0E-06 M | 58.4 | 62.7 | 60.6 |
| | | | | 3.0E-06 M | 35.5 | 34.4 | 34.9 |
| | | | | 1.0E-05 M | 19.0 | 17.4 | 18.2 |
| | | | | 1.0E-04 M | 14.8 | 13.7 | 14.3 |

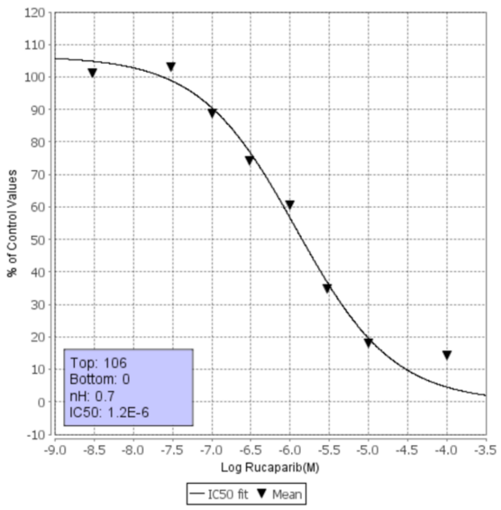

**Supplementary Figure 4** provides the raw data, in duplicate, and the dose-response curve for the interaction of rucaparib with human PIM2 kinase, as provided by the CRO (Cerep, http://www.cerep.fr/). Values are reported as percent inhibition of the control enzyme activity (see **Supplementary Methods** above). The $IC_{50}$ value calculated by non-linear regression analysis of the dose-response curve generated with mean replicate values using Hill equation curve fitting is also included as provided by Cerep.

| Cerep Compound I.D. | Client Compound I.D. | $IC_{50}$ (M) | nH | Test Concentration | % of Control Values 1st | 2nd | Mean |
|---|---|---|---|---|---|---|---|
| **Pim2 kinase *(h)*** | | | | | | | |
| 100007029-3 | Rucaparib | 7.7E-06 M | 1.0 | 3.0E-08 M | 100.7 | 86.6 | 93.6 |
| | | | | 1.0E-07 M | 90.7 | 97.3 | 94.0 |
| | | | | 3.0E-07 M | 92.1 | 86.2 | 89.2 |
| | | | | 1.0E-06 M | 94.2 | 89.1 | 91.7 |
| | | | | 3.0E-06 M | 68.0 | 70.4 | 69.2 |
| | | | | 1.0E-05 M | 36.3 | 37.1 | 36.7 |
| | | | | 3.0E-05 M | 20.3 | 19.3 | 19.8 |
| | | | | 1.0E-04 M | 12.9 | 13.4 | 13.1 |

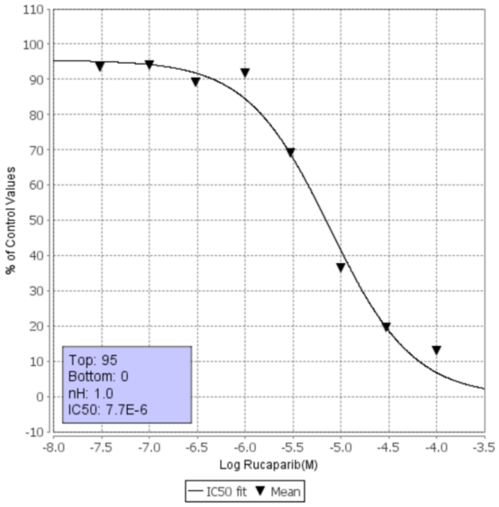

**Supplementary Figure 5** provides the raw data, in duplicate, and the dose-response curve for the interaction of rucaparib with human PRKD2 kinase, as provided by the CRO (Cerep, http://www.cerep.fr/). Values are reported as percent inhibition of the control enzyme activity (see **Supplementary Methods** above). The $IC_{50}$ value calculated by non-linear regression analysis of the dose-response curve generated with mean replicate values using Hill equation curve fitting is also included as provided by Cerep.

| Cerep Compound I.D. | Client Compound I.D. | $IC_{50}$ (M) | nH | Test Concentration | % of Control Values 1st | 2nd | Mean | Flags 1st | 2nd |
|---|---|---|---|---|---|---|---|---|---|
| **PKD2 *(h)*** | | | | | | | | | |
| 100008819-2 | Rucaparib | 9.7E-06 M | 1.0 | 3.0E-08 M | 118.0 | 106.9 | 112.4 | | |
| | | | | 1.0E-07 M | 104.3 | 108.2 | 106.2 | | |
| | | | | 3.0E-07 M | 102.2 | 102.3 | 102.2 | | |
| | | | | 1.0E-06 M | 100.7 | 98.2 | 99.5 | | |
| | | | | 3.0E-06 M | 80.0 | 89.7 | 84.9 | | |
| | | | | 1.0E-05 M | 54.9 | 50.7 | 52.8 | | |
| | | | | 3.0E-05 M | 28.7 | 29.0 | 28.8 | {}, INTER | {}, INTER |
| | | | | 1.0E-04 M | 49.6 | 43.8 | 46.7 | {}, INTER | {}, INTER |

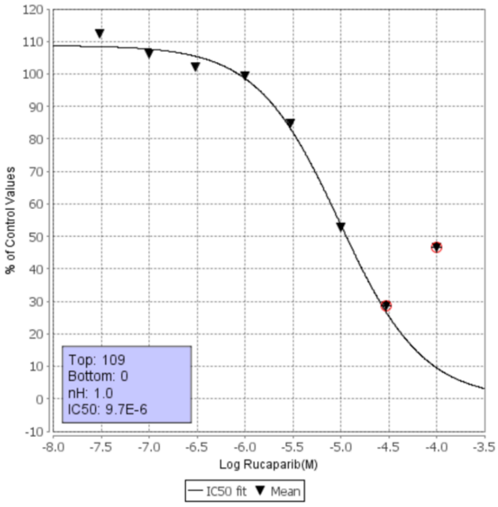

**Supplementary Figure 6** provides the raw data, in duplicate, and the dose-response curve for the interaction of rucaparib with human CDK1 kinase, as provided by the CRO (Cerep, http://www.cerep.fr/). Values are reported as percent inhibition of the control enzyme activity (see **Supplementary Methods** above). The $IC_{50}$ value calculated by non-linear regression analysis of the dose-response curve generated with mean replicate values using Hill equation curve fitting is also included as provided by Cerep.

| Cerep Compound I.D. | Client Compound I.D. | $IC_{50}$ (M) | nH | Test Concentration | % of Control Values 1st | 2nd | Mean | Flags 1st | 2nd |
|---|---|---|---|---|---|---|---|---|---|
| **CDC2/CDK1 *(h)* (cycB)** | | | | | | | | | |
| 100008819-2 | Rucaparib | 1.4E-06 M | 0.9 | 3.0E-09 M | 100.9 | 100.1 | 100.5 | | |
| | | | | 3.0E-08 M | 108.3 | 107.0 | 107.6 | | |
| | | | | 1.0E-07 M | 102.1 | 91.6 | 96.9 | | |
| | | | | 3.0E-07 M | 82.2 | 86.5 | 84.3 | | |
| | | | | 1.0E-06 M | 53.9 | 62.9 | 58.4 | | |
| | | | | 3.0E-06 M | 35.5 | 39.9 | 37.7 | | |
| | | | | 1.0E-05 M | 20.8 | 15.6 | 18.2 | | |
| | | | | 1.0E-04 M | 43.6 | 62.2 | 52.9 | {}, NSI | {}, NSI |

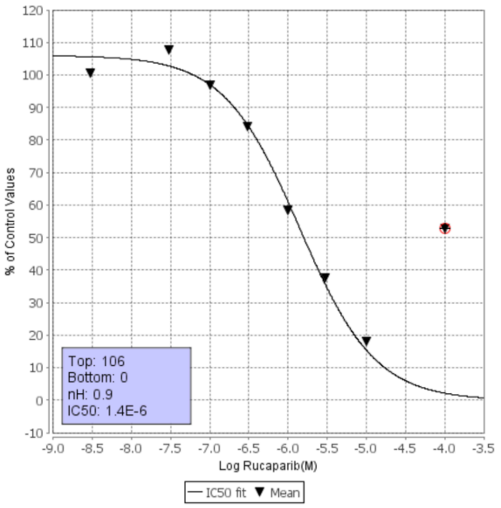

**Supplementary Figure 7** provides the raw data, in duplicate, and the dose-response curve for the interaction of rucaparib with human DYRK1A kinase, as provided by the CRO (Cerep, http://www.cerep.fr/). Values are reported as percent inhibition of the control enzyme activity (see **Supplementary Methods** above). The $IC_{50}$ value calculated by non-linear regression analysis of the dose-response curve generated with mean replicate values using Hill equation curve fitting is also included as provided by Cerep.

| Cerep Compound I.D. | Client Compound I.D. | $IC_{50}$ (M) | nH | Test Concentration | % of Control Values | | |
|---|---|---|---|---|---|---|---|
| | | | | | 1st | 2nd | Mean |
| **DYRK1a *(h)*** | | | | | | | |
| 100007029-3 | Rucaparib | 1.4E-06 M | 1.0 | 1.0E-09 M | 104.3 | 103.2 | 103.8 |
| | | | | 1.0E-08 M | 103.5 | 100.8 | 102.2 |
| | | | | 3.0E-08 M | 105.1 | 105.9 | 105.5 |
| | | | | 1.0E-07 M | 91.6 | 90.6 | 91.1 |
| | | | | 3.0E-07 M | 85.2 | 89.3 | 87.3 |
| | | | | 1.0E-06 M | 59.1 | 67.5 | 63.3 |
| | | | | 3.0E-06 M | 27.2 | 29.9 | 28.5 |
| | | | | 3.0E-05 M | 8.1 | 6.6 | 7.4 |

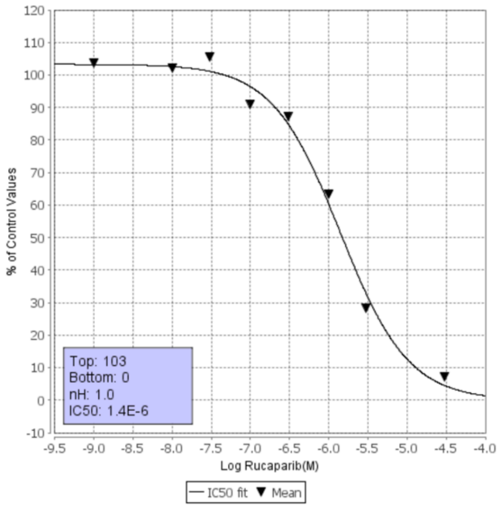

**Supplementary Figure 8** provides the raw data, in duplicate, and the dose-response curve for the interaction of rucaparib with human CDK9 kinase, as provided by the CRO (Cerep, http://www.cerep.fr/). Values are reported as percent inhibition of the control enzyme activity (see **Supplementary Methods** above). The $IC_{50}$ value calculated by non-linear regression analysis of the dose-response curve generated with mean replicate values using Hill equation curve fitting is also included as provided by Cerep.

| Cerep Compound I.D. | Client Compound I.D. | $IC_{50}$ (M) | nH | Test Concentration | % of Control Values 1st | 2nd | Mean |
|---|---|---|---|---|---|---|---|
| CDK9 *(h)* (cycT1) | | | | | | | |
| 100007029-3 | Rucaparib | 2.7E-06 M | 0.8 | 3.0E-09 M | 88.7 | 98.3 | 93.5 |
| | | | | 3.0E-08 M | 105.3 | 100.7 | 103.0 |
| | | | | 1.0E-07 M | 93.1 | 89.9 | 91.5 |
| | | | | 3.0E-07 M | 85.1 | 93.5 | 89.3 |
| | | | | 1.0E-06 M | 70.1 | 76.9 | 73.5 |
| | | | | 3.0E-06 M | 39.0 | 45.3 | 42.2 |
| | | | | 1.0E-05 M | 19.7 | 24.4 | 22.1 |
| | | | | 1.0E-04 M | 27.0 | 22.2 | 24.6 |

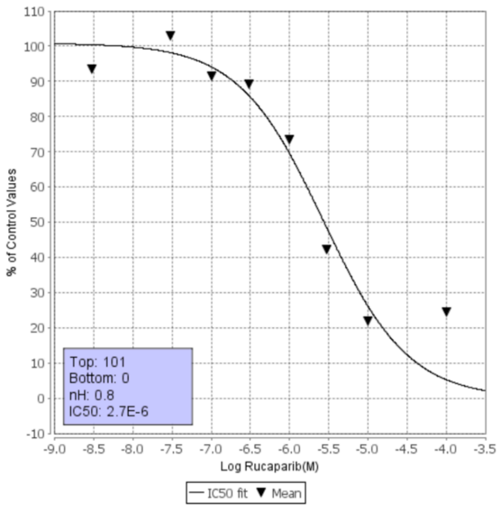

**Supplementary Figure 9** provides the raw data, in duplicate, and the dose-response curve for the interaction of rucaparib with human HIPK2 kinase, as provided by the CRO (Cerep, http://www.cerep.fr/). Values are reported as percent inhibition of the control enzyme activity (see **Supplementary Methods** above). The $IC_{50}$ value calculated by non-linear regression analysis of the dose-response curve generated with mean replicate values using Hill equation curve fitting is also included as provided by Cerep.

| Cerep Compound I.D. | Client Compound I.D. | $IC_{50}$ (M) | nH | Test Concentration | % of Control Values 1st | 2nd | Mean |
|---|---|---|---|---|---|---|---|
| **HIPK2 *(h)*** | | | | | | | |
| 100007029-3 | Rucaparib | 4.4E-06 M | 0.7 | 3.0E-08 M | 97.9 | 92.7 | 95.3 |
| | | | | 1.0E-07 M | 99.0 | 100.1 | 99.6 |
| | | | | 3.0E-07 M | 93.2 | 95.8 | 94.5 |
| | | | | 1.0E-06 M | 80.3 | 76.7 | 78.5 |
| | | | | 3.0E-06 M | 56.6 | 55.6 | 56.1 |
| | | | | 1.0E-05 M | 32.4 | 37.9 | 35.2 |
| | | | | 3.0E-05 M | 21.5 | 19.3 | 20.4 |
| | | | | 1.0E-04 M | 21.6 | 20.6 | 21.1 |

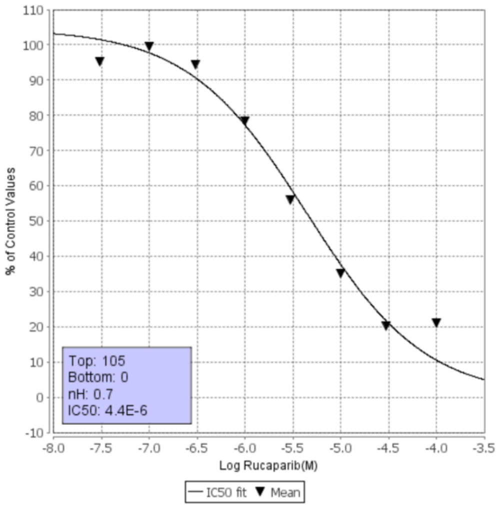

**Supplementary Figure 10** provides the raw data, in duplicate, and the dose-response curve for the interaction of rucaparib with human CK2 kinase, as provided by the CRO (Cerep, http://www.cerep.fr/). Values are reported as percent inhibition of the control enzyme activity (see **Supplementary Methods** above). The $IC_{50}$ value calculated by non-linear regression analysis of the dose-response curve generated with mean replicate values using Hill equation curve fitting is also included as provided by Cerep.

| Cerep Compound I.D. | Client Compound I.D. | $IC_{50}$ (M) | nH | Test Concentration | % of Control Values 1st | 2nd | Mean |
|---|---|---|---|---|---|---|---|
| **CK2 *(h)* (casein kinase 2)** | | | | | | | |
| 100007029-3 | Rucaparib | 7.8E-06 M | 1.0 | 3.0E-08 M | 97.4 | 91.2 | 94.3 |
| | | | | 1.0E-07 M | 85.2 | 86.2 | 85.7 |
| | | | | 3.0E-07 M | 81.9 | 75.8 | 78.8 |
| | | | | 1.0E-06 M | 94.4 | 92.7 | 93.6 |
| | | | | 3.0E-06 M | 54.7 | 69.8 | 62.2 |
| | | | | 1.0E-05 M | 37.1 | 38.1 | 37.6 |
| | | | | 3.0E-05 M | 16.5 | 15.7 | 16.1 |
| | | | | 1.0E-04 M | 16.2 | 15.6 | 15.9 |

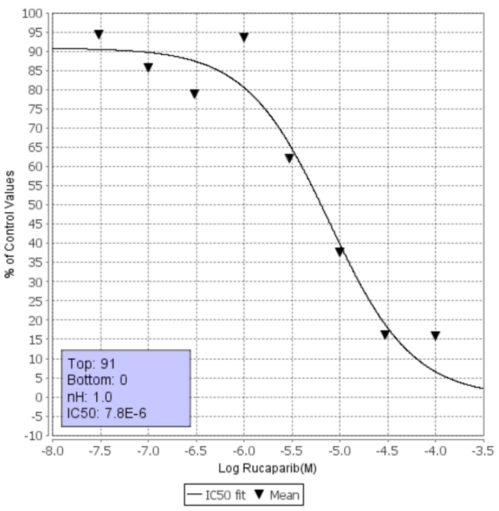

**Supplementary Figure 11** provides the raw data, in duplicate, and the dose-response curve for the interaction of rucaparib with human ALK kinase, as provided by the CRO (Cerep, http://www.cerep.fr/). Values are reported as percent inhibition of the control enzyme activity (see **Supplementary Methods** above). The $IC_{50}$ value calculated by non-linear regression analysis of the dose-response curve generated with mean replicate values using Hill equation curve fitting is also included as provided by Cerep.

| Cerep Compound I.D. | Client Compound I.D. | $IC_{50}$ (M) | nH | Test Concentration | % of Control Values 1st | 2nd | Mean |
|---|---|---|---|---|---|---|---|
| **ALK *(h)*** | | | | | | | |
| 100007029-3 | Rucaparib | 1.8E-05 M | 0.7 | 3.0E-08 M | 93.6 | 98.6 | 96.1 |
| | | | | 1.0E-07 M | 94.8 | 98.5 | 96.7 |
| | | | | 3.0E-07 M | 98.3 | 104.1 | 101.2 |
| | | | | 1.0E-06 M | 89.4 | 95.0 | 92.2 |
| | | | | 3.0E-06 M | 81.6 | 81.4 | 81.5 |
| | | | | 1.0E-05 M | 53.4 | 60.0 | 56.7 |
| | | | | 3.0E-05 M | 34.6 | 39.7 | 37.1 |
| | | | | 1.0E-04 M | 31.6 | 32.6 | 32.1 |

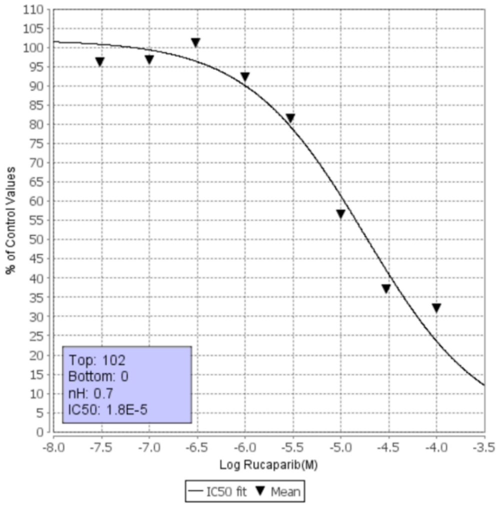

Supplement: Supplementary file 1 [file oncotarget-05-3023-s001.pdf]
